# Role of Histopathologist in Liver Transplantation

**Published:** 2017-02-01

**Authors:** B. Geramizadeh, S. A. Malek-Hosseini

**Affiliations:** 1 Department of Pathology, Shiraz University of Medical Sciences, Shiraz, Iran; 2 Transplant Research Center, Shiraz University of Medical Sciences, Shiraz, Iran; 3 *Department of Surgery and hepatobiliary Surgery and Liver Transplantation, Shiraz University of Medical Sciences, Shiraz, Iran*

**Keywords:** Liver transplantation, Liver diseases, Donor selection, pathology [Subheading], Diagnosis, Review

## Abstract

A successful liver transplantation team consists of several specialists to work closely together. The histopathologist (anatomical pathologist) is one of the key players in this multidisciplinary team. This role starts with the pre-transplantation evaluation of the recipient’s liver by diagnosis or confirming the underlying liver disease and continues with the evaluation of the explanted recipient’s liver for any further information about the underlying liver disease including malignancies such as hepatocellular carcinoma, cholangiocarcinoma, or any other incidental findings. The evaluation of the new donor liver begins with determining the suitability of the donor liver for transplantation during or before the operation and continues throughout the entire post-transplantation period by evaluating not only the allograft diseases but also evaluating other tissues for infections, malignancies, *etc*. It is worthy to note that in many of the above-mentioned situations, histopathology is the gold-standard diagnostic test. In this review, we present on various tasks of a histopathologist according to the current literature and our own experience in the largest liver transplantation center in Iran.

## INTRODUCTION

Liver transplantation is a multidisciplinary procedure needing close team work. The role played by a histopathologist is one of the crucial responsibilities in this multidisciplinary endeavor. His or her involvement sometimes starts before transplantation by reviewing the pre-transplantation liver biopsy to confirm the diagnosis and continues with donor liver biopsy evaluation to post-transplantation liver biopsies [1]. Pathologists also play a key role in evaluation and examination of the explanted livers [2]. Furthermore, there are various diagnoses of post-transplantation complications secondary to rejection or immunosuppression, inside and outside the allograft including infections and malignancies that are added to the role of a histopathologist as a member of the liver transplantation team [3].

With more than 300 liver transplantations per year, Shiraz Liver Transplant Center is the first and largest center in Iran [4, 5]. In this review we will describe our experience about the role of a histopathologist in various aspects of liver transplantation from Shiraz Liver Transplant Center and also review the experience of other centers.

**PRE-TRANSPLANTATION LIVER BIOPSY**

Decision for liver transplantation in patients with liver disease is not solely based on liver biopsy. In certain situations, the biopsy is done to confirm the primary diagnosis of the underlying liver disease and finding the primary cause of cirrhosis [6], including alcoholic or cholestatic liver diseases [7]. Sometimes, liver transplantation is done to remove an unresectable tumor, in which liver biopsy and immunohistochemistry of the tumor is necessary for the pre-transplantation diagnosis [8]. 

In our center and many other liver transplant centers, evaluation and review of the pre-transplantation liver biopsy is part of the routine pre-operative work-up. 

**EVALUATION OF THE DONOR LIVER**

Pre-existing lesions have been observed in pre-transplantation biopsies obtained as part of the assessment of potential donors for living-donor liver transplantation [9, 10]. 

One of the most important roles of a pathologist in the team is evaluation of the donor liver for its suitability for transplantation in terms of steatosis, necrosis, hepatitis, granuloma, *etc *[11]. There are different policies in liver transplant centers [2, 11-13] for evaluation of the above-mentioned conditions. In our center, for example, in every donated liver, pathological evaluation for the degree of steatosis and presence of any pathological process is performed by needle biopsy, *i.e.*, for every elective living-related case, a liver biopsy is taken and evaluated as part of the work-up. A frozen-section liver biopsy is taken from every deceased donor liver transplant and examined by a pathologist to be examined for the presence of ischemic changes, necrosis, steatosis, or any other pathologies. The findings are then discussed with the surgeons and a decision is made according to the surgeon’s gross evaluation and the pathologist’s microscopic examination of the liver [14]. In many centers, however, the decision for performing frozen section at the time of transplantation depends on the surgeon’s gross evaluation of the donor liver [12] and frozen section of the liver is only performed when the gross evaluation by the surgeon is equivocal [15, 16]. Most recent reports are in favor of doing frozen-section histological evaluation of biopsies from cadaveric (deceased) liver donors because it is an accurate, time-effective, and predictive method for the assessment of graft suitability [17, 18]. It seems that the accuracy of frozen section for evaluation of the donor liver is satisfactory in experienced hands, although some discrepancies exist that can be solved by combination of the surgeon’s and pathologist’s observations [19-21]. For living-related elective surgeries, radiologic examination (CT and MRI) are also helpful for evaluation of the steatosis in the potential donors; however, liver biopsy is still the gold-standard test [22, 23]. 

Other abnormalities that have been reported in donor biopsies include chronic hepatitis-like portal inflammation, fibrosis, iron overload, granulomas of unknown etiology, α_1_ antitrypsin globules, and amyloidosis [10].

The process of graft preservation and subsequent reperfusion, which leads to liver injury during the first 1–2 weeks following liver transplantation, can also be diagnosed and differentiated from acute rejection by time-zero donor liver biopsy [24].

**HANDLING OF THE EXPLANTED LIVER (RECIPIENT’S LIVER)**

Handling of the gross specimen of the explanted liver is a very important part of the pathologist’s responsibility in liver transplant team [25]. A thorough gross examination of liver explants typically is necessary [26]. Bread-loafing is the acceptable procedure, in which slices should be as uniformly as thin as 0.5 cm, otherwise the possibility of missed lesions will be increased, particularly small lesions. In addition, correlation with pre-operative imaging studies can be very helpful [27, 28].

There is crucial information in the explanted livers that helps hepatologists, hepatobiliary surgeons, and all the clinicians in the team of liver transplant. One of them is identification of the tumors, most importantly hepatocellular carcinomas (HCC). Helping for the accurate staging and evaluation of multifocality in patients known to have HCC is one example. The second example is finding of incidental HCCs [29]. There are also reported incidental potentially neoplastic nodules, in different conditions other than cirrhosis such as Caroli’s disease [30, 31]. Gross examination of the explanted liver for bile ducts is also very important, because presence of low-grade biliary dysplasia (BilIN) has been reported in end-stage livers even those without biliary diseases such as viral or alcoholic hepatitis [32]. In our center, incidental dual malignancies including cholangiocarcinoma and HCC have been reported in alcoholic hepatitis [33]. Other diseases such as hydatid cysts have been reported after crucial investigation of the explanted livers in patients with completely irrelevant diseases such as primary sclerosing cholangitis [34].

Another important part of gross examination is to confirm the pretransplant diagnosis of the underlying cause or find other underlying causes [35, 36].

**POST-TRANSPLANTATION LIVER BIOPSIES**

Although imaging studies and laboratory findings are important and helpful in monitoring of the transplanted liver, in many circumstances they are not sensitive enough [37]. For conditions such as rejection of the transplant, liver histology remains the gold-standard test for the diagnosis of allograft dysfunction [38, 39]. Therefore, histopathologic assessments of allograft liver biopsies have an important role in managing patients who have undergone liver transplantation [40]. 

Many of the common post-liver transplantation complications cannot be differentiated by clinical, paraclinical, and imaging studies; in many situations, more than one cause contribute to graft dysfunction, hence histopathologic assessment of allograft liver biopsies has an important role in differential diagnosis of post-transplantation complications, identifying the cause of graft damage, and subsequently initiating appropriate therapeutic intervention [40, 41].

Graft dysfunction can be caused by acute, late, and chronic rejection, recurrence of underlying diseases such as hepatitis B and C or primary biliary cirrhosis, *de novo* diseases and surgical complications, which in many conditions can be diagnosed by the pathologist in the allografted liver tissue [42].

It is also true for long-term survivors of liver transplantation to wean or decrease the immunosuppression only by the guide of histopathologic findings of the allografted liver indicating tolerance [43, 44].

**POST-TRANSPLANTATION INFECTIONS**

Post-liver-transplantation infection is one of the main causes of morbidity and mortality of transplanted patients. There are different modalities for the diagnosis of post-transplantation infections such as culture, molecular studies, *etc*; however, histopathological study has remained as a specific and complementary method for the diagnosis of many infections, especially viral and fungal infections. One of the main infections is cytomegalovirus (CMV), which can be disseminated, and involve gastrointestinal (GI) tract or the allografted liver [45, 46]. Histopathology is one of the most specific diagnostic methods for the diagnosis of CMV infection, especially when immunohistochemistry and *in situ* hybridization would be performed as well [47-49].

Other viral infections such as herpes simplex virus (HSV), Epstein-Barr virus (EBV) [50] and adenovirus can be diagnosed by pathological and immunological examination of different tissues in a liver transplant recipient [51].

Some bacterial infections (such as tuberculosis) in liver transplant recipients, have been reported to be diagnosed by pathological examination of various tissues, especially allografted liver [52]. Fungal infections are also common in liver transplant patients as immunocompromised hosts, which can be frequently diagnosed in pathological examination of various tissues such as liver, kidney, *etc* [53, 54].

**POST-TRANSPLANTATION MALIGNANCIES**

There are many adverse effects secondary to immunosuppression used in liver transplant recipients; one of these adverse effects is post-transplantation *de novo* malignancies. Nowadays, *de novo* malignancy is the second most common cause of late death after transplantation. In our center, the incidence of *de novo* malignancy has been 2.2%, which is lower than the reported studies from the West [3].

One of the most common types of malignancy reported after liver transplantation, especially in pediatric age group, is post-transplantation lymphoproliferative disease (PTLD) [55]. This disease can occur in all of the organs including the allografted liver [56, 57].

Histopathological study of the effected tissue is the gold-standard test for the diagnosis of the type of malignancy and also crucial for staging and post-surgical follow-up and chemoradiation in liver transplant recipients [58, 59].

## CONCLUSION

Liver transplantation is a life-saving procedure which needs contribution of different health professionals including a histopathologist that plays a crucial role during preoperative and post-transplantation evaluation of the liver tissue.
